# COVID-19: desperate times call for desperate measures

**DOI:** 10.1186/s13054-020-03053-8

**Published:** 2020-06-09

**Authors:** Stephan von Düring, Steve Primmaz, Karim Bendjelid

**Affiliations:** Intensive Care Unit, Geneva University Hospitals, rue Gabrielle-Perret-Gentil 4, 1205 Geneva, Switzerland

We read with interest the research letters published in *Critical Care* by Epstein et al. and Chase et al. who debate the utility of ventilator sharing in light of the COVID-19 pandemic [1, 2]. The authors argue against the fact to ventilate two patients with a single ventilator.

Citing the joint statement from the AARC on multiple patients per ventilator, the authors recommend against ventilator sharing due to the lack of control of tidal volume (*V*_T_), PEEP distribution, and pulmonary mechanic fluctuations. But are these valid arguments disqualifying concerns when demand for intubation and MV continues its upward trend? The COVID-19 pandemic has provoked a massive inflow of patients to ICUs, causing severe crisis due to the lack of equipment. As most ICU patients need intubation and MV [3], a major limitation is the lack of ventilators [4]. Some centers in Italy and the USA advocated a single ventilator for multiple patients.

The first days following intubation, patients with SARS-CoV-2 pneumonia present high thoraco-pulmonary compliance and low pulmonary recruitability [5]. This implies that alveolar overdistension is rare and *V*_T_ can safely be higher than 4–6 ml/Kg/IBW.

The objective of ventilator sharing is to save a second life by buying time to find a second ventilator. It should ideally be reserved for two patients with the same gender and similar IBW and lung mechanics, who are sedated and paralyzed. A pressure-control setting is safest as *V*_T_ will be directly dependent on the respective lung compliances of both patients. Individual flow sensors can monitor *V*_T_. Since these lungs are poorly recruitable, high PEEP is not required and PEEP distribution will not play a major role. Minute ventilation can be estimated with individual EtCO_2_ readings or PaCO_2_ measurements. Dead space or changes in lung compliance of each patient will manifest themselves by an increase in PaCO_2_-EtCO_2_ gradient. Clamping of one of the endotracheal tubes (ET) during an inspiratory pause would allow one to measure actual plateau pressure and calculate lung compliance of the other patient. Risk of cross contamination is low with the placement of high-efficiency filters at the end of each ET.

Even if intensivists are trained to tailor treatment, nothing equips them to take the grueling and unethical live-or-die choice between patients due to resource scarcity. Ventilator sharing is a rescue therapy to be considered only in specific situations for a limited time. This extraordinary procedure could save lives. Desperate times call for desperate measures.

## Data Availability

Not applicable.
